# A Markov Chain Model for Changes in Users’ Assessment of Search Results

**DOI:** 10.1371/journal.pone.0155285

**Published:** 2016-05-12

**Authors:** Maayan Zhitomirsky-Geffet, Judit Bar-Ilan, Mark Levene

**Affiliations:** 1 Department of Information Science, Bar-Ilan University, Ramat-Gan, Israel; 2 Department of Computer Science and Information Systems, Birkbeck University of London, London, United Kingdom; University of Sussex, UNITED KINGDOM

## Abstract

Previous research shows that users tend to change their assessment of search results over time. This is a first study that investigates the factors and reasons for these changes, and describes a stochastic model of user behaviour that may explain these changes. In particular, we hypothesise that most of the changes are local, i.e. between results with similar or close relevance to the query, and thus belong to the same”coarse” relevance category. According to the theory of coarse beliefs and categorical thinking, humans tend to divide the range of values under consideration into coarse categories, and are thus able to distinguish only between cross-category values but not within them. To test this hypothesis we conducted five experiments with about 120 subjects divided into 3 groups. Each student in every group was asked to rank and assign relevance scores to the same set of search results over two or three rounds, with a period of three to nine weeks between each round. The subjects of the last three-round experiment were then exposed to the differences in their judgements and were asked to explain them. We make use of a Markov chain model to measure change in users’ judgments between the different rounds. The Markov chain demonstrates that the changes converge, and that a majority of the changes are local to a neighbouring relevance category. We found that most of the subjects were satisfied with their changes, and did not perceive them as mistakes but rather as a legitimate phenomenon, since they believe that time has influenced their relevance assessment. Both our quantitative analysis and user comments support the hypothesis of the existence of coarse relevance categories resulting from categorical thinking in the context of user evaluation of search results.

## Introduction

Previous research reveals quite a high level of disagreement between the ranking of search engines and user-produced rankings [1, 2]. Inter-user agreement on ranking of search results has also been shown to be quite low due to subjectivity in human judgements [1, 3]. Scholer, Turpin and Sanderson [4] studied repeated relevance judgements of TREC (Text REtrieval Conference, http://trec.nist.gov) evaluators, and found that quite often (for 15–24% of the documents) the evaluators were not consistent in their decisions, and considered these inconsistencies to be errors made by the assessors.

In this study we investigate a scenario where users evaluate search engine results by assigning to them relevance scores more than once, at different time periods. The difference between our setting and previous experiments is that we use exactly the same query, result set and evaluation criteria in all the evaluation sessions. Thus, we create an experimental framework where the only varying parameter is the time of evaluation. The objective of this research is to provide a formal model for users’ changes in relevance assessments over time, and to utilise the model to quantify these changes, noting that we consider the “average” or “collective” behaviour of users rather than considering the change of each individual separately. As a theoretical grounding for the proposed model we make use of the theory of “coarse beliefs” [5, 6] based on the tendency of people to categorise objects into a coarse rather than fine set of categories. Examples of coarse categorisation are the star ratings given to hotels and product prices, and, in the context of this paper, the relevance judgements attached to search results. In particular, we hypothesise that users tend to distinguish between a small number of relevance categories and therefore group results according to these coarse categories. In addition, coarse beliefs often seem more natural than fine-grained beliefs when it comes to modelling human preferences, as shown by several examples in [5]. Regarding search engine results, users cannot generally distinguish between results that fall into the same coarse category, for example users may consider “relevant” results as being a coarse category, and thus, will not change their minds concerning the relevance of a results between a first round of relevance assessment and a second one occurring at a later time, despite a small, “local”, shift in opinion regarding the relevance of the result. This type of local category changes does not reflect a change in user opinion regarding the judged search result. To this end, the following change patterns are defined and explored:

1. Coarseness–according to this pattern users distinguish between a few coarse categories of relevance (following the principle of "categorical thinking" advocated by [6]) and do not perceive relevance as a continuous fine-grained range of values. This implies that, results are grouped into these categories, such that all the results inside a category are evaluated as having comparable relevance.
2. Locality–this pattern holds when change in user opinion tends to be “local”. By this we mean that, for example, that a user is more likely to change his/her relevance judgement from “relevant” to “somewhat relevant” rather than from “relevant” to “not relevant” (in accordance with the theory on modelling user preferences advocated by [6]).

To validate the above hypothesis on coarse relevance categories, we conducted a user study having overall about 120 subjects, who were asked to assess the ranking and relevance of the same set of 20 results for the same given query over two (the *initial experiment*) or three rounds (the *follow-up experiment*), three to nine weeks apart; here we concentrate only on the relevance assessments. The initial experiment was more comprehensive and contained two queries with two different result sets each, and the follow-up experiment had an additional query with a single result set. Following the third round of assessment in the follow-up experiment, the differences between the three rankings and relevancies (produced in the three rounds of the experiment) were shown to the subjects and they were asked to explain them.

More generally, we will call the initial assessment of relevance scores the first round and the subsequent assessment rounds will be called round two, round three and so on, in temporal order. At each round users judge the same set of search results under the same conditions, so it is reasonable to assume that after several rounds of assessment users will not change their minds by much, and reach a steady state. In practice an experiment will only have few rounds of assessment, typically two as in the initial experiment, or possibly three, as in the follow-up experiment, so the problem is to estimate the steady state given the available information from such experiments with a limited number of rounds.

A natural formalism for modelling users’ aggregate behaviour is that of Markov chains [7, 8], which have many applications such as the five significant ones presented in [9]. Moreover, Markov models have been widely used in the social sciences [10], and in particular in psychology to model cognitive processes [11], one prominent application being the construction of Markov models in the theory of human and animal learning [12].

Here we model the results of each experiment by a Markov chain, whose states (the coarse categories) are the relevance grades; in our case we have four relevance grades from “relevant” (grade 4) to “not relevant” (grade 1). The transition matrix of the Markov chain records the probabilities that users’ change their opinion between the rounds as to which relevance category a search result should belong to. For example, the transition from category 1 to category 2, records the proportion of users that assessed a result as being in category 1 in a given round and then changed their mind to assess the result as being in category 2 in the following round.

The transition matrices of the Markov chains will change somewhat from round to round, as users’ opinions change, and we expect a steady state to emerge after a few rounds, when users’ relevance assessments become stable and converge. Such a Markov model would be an *inhomogeneous* one, since the transition matrix changes in time [7]. However, in practice we cannot carry out the experiment until users’ opinions have converged, so as an approximation to the inhomogeneous model we propose a simpler *homogeneous* Markov model [7], where the transition matrix does not change over time. Assuming the transition matrix of the homogeneous model is *irreducible and aperiodic* (also known as one which is *ergodic*), we can compute its *stationary vector*, which in our scenario records the stable proportions of results for each relevance grade/category. The justification for the use of the simpler homogeneous Markov model is empirical, in the sense that we show that the stationary distribution does not change by much after a further round of assessment. Thus, from a practical perspective the homogenous model is reasonable, given that only few rounds can be realised in each experiment. As mentioned above we carried out two experiments, an initial one with four result sets for two queries and two rounds of assessment (and one transition matrix for each query, which we call the *round 1 transition matrix*) and a follow-up experiment with one further query and result set and three rounds of assessment (and two transition matrices for the further query, which we call the *round 1 and round 2 transitions matrices*). In order to validate the homogeneous Markov model (which from now on we will simply call the Markov model) as an approximation, we tested the transition matrices constructed from the experimental results for the following conditions:

1. The transition matrices of the Markov chains are irreducible and periodic. This implies that users’ opinions will converge.
2. The stationary distributions of the Markov chains are “close” to the empirical proportions users assign to the four categories; we use the Shannon-Jensen divergence [13], introduced below, to measure closeness. This implies that as an approximation, users’ opinions are close to convergence.
3. The stationary distributions of the Markov chains from the round 1 and round 2 transition matrices of the follow-up experiment are “close”, as are the empirical proportions users assign to the four categories induced from these matrices. This implies that the homogeneous model is a good approximation of the underlying inhomogeneous model.

We will see in the results section below that all three conditions are satisfied, providing strong evidence that the proposed Markov model is reasonable as a model of users’ change in opinion when assessing the relevance of search results.

In the context of relevance judgement the locality pattern holds, if when users change their opinions from relevance *i*, either to relevance *i-1* (unless *i = 1*) or to relevance *i+1* (unless *i = 4*). So, for example, if locality holds, then when users choose *i = 2* (“slightly relevant”), they are more likely to change their minds to *i = 1* (“not relevant”) or to *i = 3* (“somewhat relevant”), rather than to *i = 4* (“relevant”), which would be a non-local change. We can view a transition matrix which respects locality as a *nearest-neighbour* matrix where non-local transitions have probability zero; in technical terms such a matrix, defined below, is called a *tri-diagonal matrix*; we call a Markov chain with a tri-diagonal transition matrix a *tri-diagonal Markov chain*.

In order to validate locality in the context of the Markov model, we construct tri-diagonal matrices counterparts for each transition matrix, by setting the non-local transition probabilities to zero and renormalising the resulting matrix so that the row probabilities add up to one. We then test the tri-diagonal matrices against their counterparts for the following conditions:

1. The proportion of transitions in the tri-diagonal matrices relative to their counterpart transitions matrices is very high. This implies that most changes in relevance assessments are local.
2. The stationary distributions of the tri-diagonal Markov chains are “close” (in the Shannon-Jensen sense) to the empirical proportions users assign to the four categories according to these tri-diagonal transition matrices. This implies that users’ local opinion changes are close to convergence.
3. The stationary distributions of the tri-diagonal Markov chains are “close” (in the Shannon-Jensen sense) to the stationary distribution of their Markov chain counterparts. This validates modelling users’ change of opinion with the tri-diagonal Markov chains.

We will see in the results section below that all three conditions are satisfied to a high degree in the data collected from our experiments, providing strong evidence for the locality pattern.

The two main contributions of the paper are; (i) presenting a viable model for users’ change in relevance assessments over time, and (ii) making use of this model to argue that users’ change of opinion is local. User preference change in assessment of search results has been widely studied in the literature [14]. However, we stress that this is a first study that investigates the factors that cause changes in users’ relevance and ranking judgements when the only variable that is different in every assessment round is time. As a further validation of our hypothesis, analysis of users’ comments from the follow-up experiment on the changes they performed in their judgments in the three evaluation sessions reveals that the main factors for the changes in user assessment of search results over time were categorical thinking, knowledge acquisition, self-criticism and the emotional state of a user.

The rest of the paper is organised as follows. In the second section, we provide the related literature review, and in the third section, we present our methodology including the utilisation of the Markov model for users’ changing their minds when assessing search engine results. In the fourth section, we evaluate the Markov model on the data we collected from the user experiments, and finally, in the fifth section, we give our concluding remarks.

## Related Work

We first review previous research that studied user relevance and ranking assessment behaviour, and then describe experiments that investigated the change in user assessment of search results. Studies of user assessment of ranked search results found that users are usually interested in only 10–20 items displayed on the first results page [15, 16]. It has been shown that the top search engine results are presumed to be the most relevant for the given query [15]. In the experiments with Google rankings assessment for 34 different queries composed and judged by the same subject, the fifth ranked result was judged to be of highest relevance slightly more than the top ranked result [16]. In addition, as indicated by the results in [1, 2] there is a quite a high level of disagreement between the ranking of search engines and user-produced rankings [1, 2]. In a study in 2007 [1] users were presented with randomly ordered result sets retrieved from Google, Yahoo! and MSN (now Bing) and were asked to choose and rank the top-10 results. The findings showed low similarity between the users and the search engines rankings. In a follow-up study [2], country-specific search results were tested in a similar way. In this case it was shown that at least for Google, the users preferred the results and the rankings of the geographically local Google version over other versions. As opposed to the current study these studies only asked the users to rank the results, without asking for their relevance judgements, and users were asked to rank the results only once. In the experiments reported in [1, 3] inter-user agreement on ranking of search results has also been shown to be quite low due to subjectivity in human judgements.

Most previous work investigated the dynamics and changes in relevance evaluation during an iterative search process, when the information need, relevance judgment criteria, query terms and result sets change due to information gained in the previous search iterations [14]. One of the first studies of such changes was by Rees and Schultz [17]. Saracevic [14] reviews additional studies, where the relevance assessments at different points in the information seeking task of more than two participants were investigated [18–25]. However, the setting of the above mentioned studies is different from the current study’s setting. In the previous studies the users’ information need and evaluation criteria changed as the task evolved. In the current study, each round was carried out as a separate closed evaluation session, and the participants were explicitly instructed to use the same criteria and goals, the same query and result sets in all the rounds of the experiment. Hence, the question we address is what happens in more than one standalone evaluation rounds (sessions) when the tasks are identical, and assessed at different points in time? The only difference between the rounds is that, when doing the consecutive rounds, users have already been exposed to a set of documents (or their snippets) once before in the previous session.

A similar type of scenario was examined by Scholer et al. in [4]. They compared repeated relevance judgements of the same TREC documents and queries by the same assessors on a 3-point scale. They found that the assessors were not consistent in their decisions for 15–24% of the documents, and explained these inconsistencies as errors made by the assessors. Scholer et al. [26] asked their users to evaluate the relevance (on a 4-point scale) of three documents twice, as part of a larger scale experiment. They inserted “duplicate” results within a single evaluation session to test the influence of the “temporal” factor, where the total time for an evaluation session was approximately one hour. In their experiment only 50% self-agreement was attained.

As opposed to the previous studies, the documents judged in our experiments were search engine results from the Web, and in addition, we measured changes in users' judgments rather than in experts' judgements. We believe that the changes in ranking and relevance judgements are not necessarily errors and explore factors that might cause such changes. Apart from measuring the change in user assessment we also explore the novel research question of whether the changes stabilise in time.

## Empirical Evaluation

### Experimental setup

We conducted a user study with three groups of subjects who were all information science students. The students were all presented with the following scenario: “Your aim is to learn about the given topic based only on the search results, in order to be able to prepare a good summary of the topic”. The two queries presented to the first group of 42 students and to the second group of 41 students were Alzheimer’s syndrome (in Hebrew) and Big Data (in English). For group one two result sets were created, the first based on the Alzheimer’s syndrome query Google results displayed on the first and the tenth result page (i.e. results 1 to 10 and 101 to 110), and the second based on the Big data query from Google and Bing results which were displayed on the first page for each of the search engines. For the second group the first result set was based on Big Data query results from Google and Bing’s first result pages, and the second result set was from the Google first and tenth pages. We denote these four result sets as Q1, Q2, Q3 and Q4 throughout the paper. The query topic for the third group of 35 students was presented with was “Cyber warfare” (in English). For this third group the result set comprised the Google results displayed on the first and the tenth result pages. The participants were supposed to possess some basic background knowledge of these topics that were selected due to their high actuality. However, these topics were not part of the students’ course of study. The subjects were then presented with a randomly ordered list of 20 search results for the given query. The search results were presented in a similar style to SERPs (Search Engine Results Pages), i.e. title, URL and snippet as displayed by the search engine. The users were asked to assess the relevance of all 20 items on a four level ordinal scale: “not relevant” (1), “slightly relevant” (2), “somewhat relevant” (3) and “relevant” (4). Google Forms were used to collect the answers.

As part of the initial experiment the first two groups of subjects conducted two rounds of result assessment with four different result sets for the two queries above, while the second round took place two months after the first round. The follow-up experiment was conducted by the third group only, with three weeks between the first and the second rounds and with two months between the second and the third assessment rounds. For all the groups and experiments during the first, second and third rounds of the experiments participants were given exactly the same instructions, and exactly the same query and set of search results, but these were displayed in a different random order, to avoid participants just copying their previous assessments. In each round all the participants saw the results in the same random order. For the third group after the third session of the follow-up experiment, the subjects were exposed to the differences in their judgements and were asked to explain them. They returned their feedbacks five weeks after completing the follow-up experiment.

To mitigate for presentation bias [27] the participants were instructed to evaluate every result independently of its position in the list. In addition to relevance judgments, subjects were asked to rank the ten best results where no ties were allowed. To check whether there was still a presentation bias in our data we calculated the aggregated ranking by summing up these ranks assigned by all the subjects in the group for each result. Then, the results were sorted in the ascending order of the aggregated ranks and ranked accordingly from 1 to 20. The presentation order ranking was created by numbering the results according to their display order in the given round. Spearman’s rank correlation (*r*_*s*_) was then computed between the aggregated ranking and the presentation order ranking.

As can be viewed from Table 1 the only case (*) where there was a significant correlation (and thus there may have been some presentation bias) is the Alzheimer Google&Bing result set from round 1. We noticed that for this set there was a larger number of highly relevant results (according to the subjects’ judgments) than for the other result sets, and thus it might have been harder for the subjects to make a decision on them.

**Table 1:** Spearman's correlation between the aggregated ranking and presentation order for different queries and results sets.

Query & Result Set	For round 1		For round 2		For round 3	
	*r*_*s*_	*p value*	*r*_*s*_	*p value*	*r*_*s*_	*p value*
Bigdata Google&Bing	-0.11	0.65	0.00	1.00	n/a	n/a
Bigdata Google10&100	-0.12	0.60	0.08	0.74	n/a	n/a
Alzheimer Google10&100	0.35	0.14	-0.13	0.57	n/a	n/a
Alzheimer Google&Bing	0.64	0.01*	0.16	0.49	n/a	n/a
Cyber Warfare	0.10	0.67	0.39	0.09	0.23	0.33

* The case where there was a significant correlation (and thus there may have been some presentation bias).

The ranking and relevance judgment tasks were part of the participants’ class assignment. They were informed that their rankings and relevance judgements will be aggregated and analysed anonymously, and those who wished not to contribute their data to the aggregated study were asked to inform the class instructor by email. They were also told that this decision had no effect whatsoever on their grades. No students asked to withdraw their data. Although the experiment involves human subjects (students), no personal information was gathered on them. The Faculty of Humanities’ IRB (ethics committee) waived the need for written consent. There were no minors enrolled in the study. The IRB of the Faculty of Humanities at Bar-Ilan University approved the experiment.

### Patterns of categorical thinking for user assessment of search results

To analyse the results of the experiments we investigate whether two types of patterns of user behaviour from coarse beliefs theory hold for the case of evaluation of search results: (1) coarseness and (2) locality.

Our relevance evaluation process creates four groups of results according to their relevance grade. Thus, the type of coarse categories we define and use in this study is based on the relevance grades, which seems to be natural. Note that here we are capturing the average user behaviour through statistics collected from a group of users, which may be viewed as an application of the “wisdom of the crowds” [28].

### A Markov chain model for users’ change in relevance assessments

We now formalise our Markov chain model for measuring the aggregate change in users’ assessments between the rounds of the experiment under consideration. We first present the Markov chain preliminaries needed for the formalism, and then discuss how we use the model to assess users’ change in assessments in light of the methodology described in the introduction.

We briefly review the concepts pertaining to Markov chains [7, 8], needed for the development of our user model. A (homogeneous) Markov chain (or simply a Markov chain) *M = (S*, *P)* consists of states, *i*, in a finite set *S* of size *n*, and an *n*-by-*n* transition matrix = {*p*_*ij*_ | *i*, *j* ∈ *S*}. The transition matrix *P* is row stochastic, i.e. for all states, *i*, ∑_*j*_ *p*_*ij*_ = 1, and we assume that *P* is *irreducible and aperiodic* (also known as one which is *ergodic*), i.e. there exists *m* such that *P*^*m*^ > 0. Ergodicity implies that the Markov chain has a stationary distribution, *π* such that = *π*. We note that a Markov chain also has an initial distribution, however, since in our case the chain is irreducible and aperiodic, the stationary distribution is independent of the initial distribution [7, 8].

As explained in the introduction we make use of the Markov chain to model how a group of users’ changes their mind from one round, say *k*, to the next one, say *k+1*, recalling that the initial experiment has two rounds (giving rise to the round 1 transition matrix) and the follow-up experiment has three (giving rise to the rounds 1 and 2 transition matrices). As the states are relevance grades, the transitions *p*_*ij*_ capture the probabilities that users change their mind regarding to which relevance category a search result belong to; *p*_*ii*_ thus denotes the probability that the users do not change their minds regarding category *i*.

A *tri-diagonal matrix* [29] is a square matrix having nonzero elements on the main-diagonal, the super-diagonal above it and the sub-diagonal below it, and zeros elsewhere. A Markov chain whose transition matrix is tri-diagonal captures the notion of nearest-neighbour transitions, and can easily be shown to be irreducible and periodic for homogeneous Markov chains. (We note that Markov chains that are characterised by tri-diagonal transition matrices are also known as birth-and-death Markov chains [30].)

Given a transition matrix *P*, we may project it onto a tri-diagonal matrix by replacing all the nonzero elements in the matrix, apart from those on the main, sub and super diagonals, by zeros, and then by renormalising the matrix we obtain a tri-diagonal matrix. We denote this transformation by *τ(P)*, which returns the null matrix whenever this transformation is not possible, i.e. when the transformed matrix is not tri-diagonal; for the rest of the paper we will assume that *τ(P)* is not null.

The Jensen-Shannon divergence (*JSD*) [13] is a nonparametric measure of the similarity between two distributions *{p*_*i*_*}* and *{q*_*i*_*}*, where *i = 1*, *2*,…, *n*. The JSD is a well-known statistic for goodness-of-fit testing, having the same asymptotic distribution as the well-known Pearson chi-squared test [31].

The formal definition of the JSD, which is a symmetric version of the Kullback-Leibler divergence, based on Shannon’s entropy [32], is given by:

$$\text{JSD}\left(\left\{p_{i}\right\}\left\{q_{i}\right\}\right)=1-\sqrt{\frac{1}{2ln2}\sum_{i=1}^{n}\left(p_{i}\;ln\frac{2p_{i}}{p_{i}+q_{i}}+q_{i}\;ln\frac{2q_{i}}{p_{i}+q_{i}}\right)},$$

where *ln*, represents the natural logarithm, and we use the convention that 0*ln*0 = 0. We note that the factor of 1/(2 *ln* 2) in the square root term is in order to normalise the JSD, and that JSD({*p*_*i*_}{*q*_*i*_}) = 1 when the two distributions coincide.

## Results and Analysis

We first analyse the follow-up experiment as it contained three rounds of relevance judgments, as opposed to only two rounds for the initial experiment, and only then present the results from the initial experiment to add weight to the results. We note the initial and follow-up experiments were completely independent as there was no overlap between the users of the two experiments, and, in addition, the users in the follow-up experiment did not have any knowledge of the initial one. For the purpose of the analysis, we constructed a *round1 frequency matrix* from the first and second relevance assessment rounds, which is a 4x4 matrix recording the number of times *f*_*ij*_ that users changed their minds by assigning a search result grade *i* in the first round and grade *j* in the second; similarly, we construct the *round 2 frequency matrix* from the round two and round three relevance assessments. The frequency matrices for rounds 1 and 2 are shown, respectively, on the right-hand (rhs) and left-hand sides of Table 2 where *R* stands for relevance grade. The projections of the frequency matrices shown in Table 2 onto tri-diagonal frequency matrices, to capture the local (nearest-neighbour) changes, are shown on the rhs and lhs, respectively, of Table 3.

**Table 2:** Round 1 (lhs) and round 2 (rhs) 4x4 frequency matrices for relevance judgments.

*R*	1	2	3	4	*R*	1	2	3	4
**1**	104	35	12	11	**1**	116	32	9	11
**2**	44	94	40	22	**2**	32	105	36	13
**3**	12	38	42	35	**3**	6	40	50	26
**4**	8	19	28	156	**4**	12	23	36	153

**Table 3:** Round 1 (lhs) and round 2 (rhs) frequency matrices for relevance judgments projected onto tri-diagonal frequency matrices.

*R*	1	2	3	4	*R*	1	2	3	4
**1**	104	35	0	0	**1**	116	32	0	0
**2**	44	94	40	0	**2**	32	105	36	0
**3**	0	38	42	35	**3**	0	40	50	26
**4**	0	0	28	156	**4**	0	0	36	153

The transition matrices for rounds one and two, obtained by normalising the ones shown on the rhs and lhs of Table 2 are shown, respectively, on the rhs and lhs of Table 4, and can be seen to be irreducible and periodic. Correspondingly, the matrices resulting from normalising the projection of the original frequency matrices onto tri-diagonal frequency matrices, shown on the rhs and lhs of Table 3, are shown, respectively, on the rhs and lhs of Table 5, and are also seen to be irreducible and periodic. The stationary distributions of the transition matrices in Tables 4 and 5 are shown in Table 6.

**Table 4:** The transition matrices obtained by normalising the round 1 (lhs) and round 2 (rhs) frequency matrices shown in Table 2.

*R*	1	2	3	4	*R*	1	2	3	4
**1**	0.6420	0.2160	0.0741	0.0679	**1**	0.6905	0.1905	0.0536	0.0655
**2**	0.2200	0.4700	0.2000	0.1100	**2**	0.1720	0.5645	0.1935	0.0699
**3**	0.0945	0.2992	0.3307	0.2756	**3**	0.0492	0.3279	0.4098	0.2131
**4**	0.0379	0.0900	0.1327	0.7393	**4**	0.0536	0.1027	0.1607	0.6830

**Table 5:** The transition matrices obtained by normalising the round 1 (lhs) and round 2 (rhs) tri-diagonal frequency matrices shown in Table 3.

*R*	1	2	3	4	*R*	1	2	3	4
**1**	0.7482	0.2518	0	0	**1**	0.7838	0.2162	0	0
**2**	0.2472	0.5281	0.2247	0	**2**	0.1850	0.6069	0.2081	0
**3**	0	0.3304	0.3652	0.3043	**3**	0	0.3448	0.4310	0.2241
**4**	0	0	0.1522	0.8478	**4**	0	0	0.1905	0.8095

**Table 6:** Stationary distributions, denoted by π, for the transition matrices in Tables 4 and 5.

*π*	*R*/Table	1	2	3	4
***π***_**12**_	lhs of Table 4	0.2349	0.2501	0.1693	0.3457
***π***_**23**_	rhs of Table 4	0.2469	0.3116	0.1924	0.2491
***π***_***12***_***τ***	lhs of Table 5	0.2441	0.2486	0.1691	0.3382
***π***_***23***_***τ***	rhs of Table 5	0.2699	0.3155	0.1904	0.2241

We can now validate the homogeneous Markov model. Firstly, as noted above the transition matrices in Table 4 are irreducible and periodic, and thus users’ judgements will converge after a certain number of rounds. Secondly, the JSD between the stationary distributions of these matrices, shown in the first two rows of Table 6, and the normalised proportions users assigned to the relevance categories on rounds 1 and 2, shown on the rhs of Table 7, are given by

JSD(π12,P1)=0.9760andJSD(π23,P2)=0.9597,

implying that *P1* and *P2* are close to the stationary distributions and thus users judgements are close to convergence. Thirdly, the JSD between the stationary distributions of the Markov chains from the round 1 and round 2 transition matrices, shown in the first two rows on Table 6, and JSD between the normalised user proportions shown on the rhs of Table 7, are given by

JSD(π12,π23)=0.9064andJSD(P1,P2)=0.9689,

implying that the homogeneous Markov model is a good approximation of the underlying inhomogeneous model described in the introduction, since users judgements are close to convergence and do not change by much between the rounds. We observe that this statistic is provided solely from the follow-up experiment, which had three rounds of assessment, as opposed to the initial experiment which had only two rounds of assessment for each query. Overall these results provide strong evidence for the homogeneous Markov model as one describing how user judgements converge to a stationary distribution.

**Table 7:** Proportion of search results assigned to each relevance category after the first and second rounds (lhs) and their normalisations (rhs), denoted by P1 (first row) and P2 (second row).

*R*	1	2	3	4	*R*	1	2	3	4
**1**	166	186	122	224	**1**	0.2400	0.2657	0.1743	0.3200
**2**	168	200	131	203	**2**	0.2371	0.2857	0.1871	0.2900

We now turn to the validation of the locality pattern from the follow-up experiment. We first see in Table 8 that the tri-diagonal frequency matrices, shown in Table 2, capture about 90% of users’ changes, shown in the frequency matrices of Table 2 from which the tri-diagonal matrices are derived, indicating that most of users’ changes in relevance assessment between the rounds are indeed local.

**Table 8:** Proportion of frequencies in the tri-diagonal matrices for relevance judgments.

Round	Tridiagonal	Original	Proportion
**1**	616	700	0.8800
**2**	626	700	0.8943

Secondly, the JSD between the stationary distributions of the tri-diagonal matrices, and between the normalised user proportions shown on the rhs of Table 7, are given by

$$JSD\left({\pi 12}^{\tau }\;,\;P1\right)\;=\;0.9793\;\text{and}\;JSD\left({\pi 23}^{\tau }\;,\;P2\right)\;=\;0.9339,$$

indicating that the local judgements of users are close to convergence as modelled by the tri-diagonal nearest-neighbour matrices.

Thirdly, the JSD between the stationary distributions of the original transition matrices, shown in Table 4, and the projected tri-diagonal matrices, shown in Table 5, are given by

$$JSD\left(\pi 12,\;\pi \;12^{\tau }\;\right)\;=\;0.9903\;\text{and}\;JSD\left(\pi 23,\;\pi \;23^{\tau }\;\right)\;=\;0.9706,$$

indicating that modelling users’ change in relevance judgements is well-modelled by the tri-diagonal matrices conveying the local changes made by these users.

We now provide further evidence of locality of user judgement from the initial experiment by summarising the results for the four result sets in Table 9, noting that all the induced transition matrices are irreducible and periodic. In row 1 we show the JSD between the stationary distributions of the transition matrices for the four result sets and the normalised proportions users assigned to the relevance categories. Then, in row 2 we show the JSD between the stationary distributions of the corresponding tri-diagonal transition matrices and the normalised proportions users assigned to the relevance categories. Finally, in row 3 we show the JSD between the stationary distributions of the transition matrices and the corresponding stationary distributions of the respective tri-diagonal transition matrices. It can be seen that the similarities for the initial experiment, as measured by JSD, are comparable to those we reported above for the follow-up experiment. Moreover, the proportions shown in Table 10, show, that the tri-diagonal frequency matrices capture about 90% of users’ changes for the initial experiments, as was also shown for the follow-up experiment above.

**Table 9:** JSD results for the four result sets of the initial experiment and their averages.

*JSD*	Q1	Q2	Q3	Q4	Average
**1**	0.9841	0.9855	0.9540	0.9845	0.9770
**2**	0.9842	0.9000	0.8971	0.9697	0.9287
**3**	0.9621	0.9037	0.9426	0.9726	0.9453

**Table 10:** Proportion of frequencies in the tri-diagonal matrices for the relevance judgments of the four result sets.

Q1	Q2	Q3	Q4	Average
0.8744	0.8598	0.8964	0.8695	0.8750

In summary the results of the initial experiment, confirm the results from the follow-up experiment, that the homogenous model is suitable for modelling users’ change in relevance judgements over time, and that the locality pattern, a hallmark of categorical thinking, holds, implying that users’ changes are indeed local.

Finally, in the next subsection we provide a content analysis of the subjects’ feedback on the changes in their relevance and ranking assessments in different rounds of the experiment. Our objective in this analysis was to learn the main reasons for these changes in subjects’ assessments and examine whether they reinforce the quantitative analysis of these assessments presented above.

### Analysis of the user feedback on the changes in their result assessment

As shown in the preliminary study [33], the analysis of the answers that our subjects provided to explain the changes in their assessments of the search results, reveals that most users believe that time influences human relevance judgements. In their feedback, the users related both to ranking of the top-10 out of the 20 results and to relevance judgment of each of the 20 results. Only a few were surprised and confused by seeing the differences in their own judgements in different rounds of the experiment. Four out of 35 subjects from the follow-up experiment assessed their judgements as consistent with a few local changes between close ranks. They explained that is was due to adhering to the same criteria and type of thinking across all three rounds of the experiment.

The majority of the subjects viewed as most important the binary division between top-10 (ranked) and last-10 (unranked/non-relevant) result subsets, resulting in two coarse categories. The changes of ranks within these two subsets were considered as local and insignificant by the subjects: “*First of all*, *I notice that in spite of the changes in ranks between the rounds*, *I included the same results in the top-10 ranks*, *of course*, *without preserving the previous order within the top-10 results*”. This is an explicit argument for categorical thinking [6] of the subjects when ranking search results, showing the locality of changes and the relative stability of top-10 and last-10 subsets. Actually, we found, as demonstrated by the quantitative analysis above, that coarse categories theory holds even for more fine-grained categories of relevance (four categories rather than two), as assessment changes were mostly within the coarse categories.

In particular, the following repetitive factors have been detected as causing the change in user judgements:

1. categorical thinking–binary division of relevant vs. non-relevant, the local changes of ranks within the relevant groups are not significant for users: “*In most cases there was no significant differences in my judgements–most of the unranked results in the first round remained unranked in the next rounds as well*, *and most of the ranked results were around the same ranks in all the three rounds*.”;
2. self-improvement–correcting the mistakes from the first rounds or trying to improve the ranking in every next round: “*I acted differently in every round of the experiment*, *and the reason for that is the feeling that there is something tricky about ranking the results and I have to try and improve my way of ranking every time*.”;
3. knowledge acquisition–during the first rounds of the experiment the subjects were exposed to information on the topic, which caused them in later rounds to rank the results differently: “*The reason for differences in my rankings is that in the beginning of the experiment I only had a very general idea on the topic while from round to round my knowledge expanded and thus my ranking had changed accordingly*.”. However, even in this case, most of the changes in the relevance assessments for these users were small and local, as measured by the closeness of the stationary distributions from consecutive rounds.
4. emotional factors that influence the subjective assessment of relevance, which depend on place (of doing the ranking), environment, mood, focus/anxiety and other emotions: “*The only way to explain this exceptional phenomenon is because the human ranking is influenced by many factors*, *such as the mood*, *the anxiety*, *the loss of patience*, *the external stimulus*, *even the design of the website*, *the place where I have done the rankings (the first and the third rounds were conducted at the campus while the second one was at home)*.”.

## Conclusion

The two main contributions of the paper are; (i) it presents a testable Markov chain model for users’ change in relevance assessments over time, and (ii) it makes use of this model to quantify the assertion that users’ change of opinion is local. The results provide strong evidence for both the viability of the Markov chain model and the locality pattern in users’ opinion changes over time. In addition, to support the quantitative evidence for categorical thinking, the experimental design included a user feedback, qualitative stage, where users were shown the changes in their own assessments and had to provide their opinion about them. The analysis of the user feedback indicates that users indeed think in categories and consider local changes insignificant. The other factors for change in judgment were the influence of the information learnt on the topic, the tendency to self-criticism and improvement of the ranking, and the change in the emotional state of the subjects.

As far as we are aware of, this is the first study that provides a theoretically grounded model that shows evidence for the existence of coarse relevance categories and categorical thinking in users’ perception of search results. This is also a first study that investigates factors in users’ perception of influence of time on change in their relevance assessments.

In ongoing research we have conducted a preliminary experiment, where a group of users had to choose the optimal number of relevance categories for the same 20 search results, and then assign each result to a category. The average number of categories chosen was 4.1, which led us to the decision to use a 4-point scale for relevance in these experiments. Investigating whether the changes in user judgements are local and users’ behavior is stationary in the setting where the number of categories is not predetermined is a subject for future work.

As a closing remark we mention that this research may have practical implications for personalisation, as users' preferences change over time and therefore the ranking of search engine results should adapt to this.
